# Maternal thyroid function and child educational attainment: prospective cohort study

**DOI:** 10.1136/bmj.k452

**Published:** 2018-02-20

**Authors:** Scott M Nelson, Caroline Haig, Alex McConnachie, Naveed Sattar, Susan M Ring, George D Smith, Debbie A Lawlor, Robert S Lindsay

**Affiliations:** 1 School of Medicine, University of Glasgow, Room 2.52 Level 2, New Lister Building, Glasgow Royal Infirmary, Glasgow G31 2ER, UK; 2 National Institute for Health Research, Bristol Biomedical Research Centre, Bristol, UK; 3 Robertson Centre for Biostatistics, University of Glasgow, Level 11, Boyd Orr Building, Glasgow, UK; 4 Institute of Cardiovascular and Medical Sciences, British Heart Foundation Glasgow Cardiovascular Research Centre, University of Glasgow, Glasgow, UK; 5 The Medical Research Council Integrative Epidemiology Unit at the University of Bristol, Oakfield House, Bristol, UK; 6 Population Health Science, Bristol Medical School, University of Bristol, Oakfield House, Bristol, UK

## Abstract

**Objective:**

To determine if first trimester maternal thyroid dysfunction is a critical determinant of child scholastic performance and overall educational attainment.

**Design:**

Prospective cohort study.

**Setting:**

Avon Longitudinal Study of Parents and Children cohort in the UK.

**Participants:**

4615 mother-child pairs with an available first trimester sample (median 10 weeks gestation, interquartile range 8-12).

**Exposures:**

Free thyroxine, thyroid stimulating hormone, and thyroid peroxidase antibodies assessed as continuous measures and the seven clinical categories of maternal thyroid function.

**Main outcome measures:**

Five age-specific national curriculum assessments in 3580 children at entry stage assessment at 54 months, increasing up to 4461 children at their final school assessment at age 15.

**Results:**

No strong evidence of clinically meaningful associations of first trimester free thyroxine and thyroid stimulating hormone levels with entry stage assessment score or Standard Assessment Test scores at any of the key stages was found. Associations of maternal free thyroxine or thyroid stimulating hormone with the total number of General Certificates of Secondary Education (GCSEs) passed (range 0-16) were all close to the null: free thyroxine, rate ratio per pmol/L 1.00 (95% confidence interval 1.00 to 1.01); and thyroid stimulating hormone, rate ratio 0.98 (0.94 to 1.02). No important relationship was observed when more detailed capped scores of GCSEs allowing for both the number and grade of pass or when language, mathematics, and science performance were examined individually or when all educational assessments undertaken by an individual from school entry to leaving were considered. 200 (4.3%) mothers were newly identified as having hypothyroidism or subclinical hypothyroidism and 97 (2.1%) subclinical hyperthyroidism or hyperthyroidism. Children of mothers with thyroid dysfunction attained an equivalent number of GCSEs and equivalent grades as children of mothers with euthyroidism.

**Conclusions:**

Maternal thyroid dysfunction in early pregnancy does not have a clinically important association with impaired child performance at school or educational achievement.

## Introduction

Overt and subclinical thyroid diseases affect up to 10% of pregnancies depending on the laboratory reference range used.^1^ Thyroid hormone is essential for normal brain development, with both congenital thyroid deficiency and excessive exposure during fetal life associated with long term cognitive impairment.^2^ ^3^ As the developing fetus is dependent on maternal thyroid hormones until the late first trimester,^4^ profound untreated maternal hypothyroidism in early pregnancy has been associated with an overall seven point reduction in child intelligence quotient.^5^ Maternal subclinical hypothyroidism and isolated hypothyroxinaemia have also been associated with impairments in child intelligence quotient,^2^ ^5^ ^6^ ^7^ arithmetic skills,^8^ scholastic performance,^9^ and motor skills,^5^ ^7^ as well as poorer reaction time,^10^ delays in attention,^5^ and increased attention deficit hyperactivity disorder symptoms.^11^ Maternal subclinical thyroid dysfunction has also been associated with an increased risk of miscarriage, gestational hypertension, pre-eclampsia, gestational diabetes, and preterm birth,^12^ ^13^ and that treatment in some but not all studies improved outcomes,^14^- ^16^ has prompted calls for universal thyroid function screening to facilitate early identification and treatment of both overt and subclinical disease.^17^ ^18^ This is despite appropriately powered trials demonstrating that intervention with maternal levothyroxine treatment is not effective at notably improving perinatal or childhood cognitive outcomes for women with subclinical thyroid dysfunction.^19^ ^20^ ^21^

Indeed, a large prospective randomised controlled trial which screened 21 846 mothers did not show any difference in intelligence quotient at age three between children of mothers with reduced thyroid function who were treated with levothyroxine or not.^21^ Similarly, a more recent trial which screened 97 228 pregnant women showed that levothyroxine treatment of maternal subclinical hypothyroidism or isolated hypothyroxinaemia had no benefit on neurodevelopmental scores or cognitive function through to age five.^20^ Furthermore, screening and treatment of reduced maternal thyroid function did not alter birthweight or the incidence of preterm birth,^20^ ^21^ which confirms current obstetric guidelines to not perform routine antenatal screening for hypothyroidism in pregnancy.^22^ ^23^ Not all professional bodies agree with continued endorsement of early initiation of treatment.^12^ ^18^ That these trials initiated treatment at a time when the fetal thyroid is functional, and did not stratify relative to thyroid peroxidase antibody status, has led to the suggestion that treatment was too late to be effective and not targeted appropriately. The trials were limited to the assessment of young children and used different measures of cognitive function, contributing to the lack of clarity.

Individual and overall cognitive domains have been shown to be predictive of educational attainment,^24^ ^25^ ^26^ with the latter recognised as an important determinant of lifelong health, child and adult mortality, personal wealth, and national economic success.^27^ ^28^ Assessment of first trimester maternal thyroid function in samples from the index pregnancy combined with long term follow-up of children with educational outcomes would address many of the criticisms levelled at the trials.^29^ ^30^ ^31^ ^32^ We therefore examined, in the prospective Avon Longitudinal Study of Parents and Children cohort, if maternal gestational thyroid function is associated with child performance in repeat national standardised educational assessments through to the age of leaving secondary school.

## Methods

### Participants

**Fig 1:**
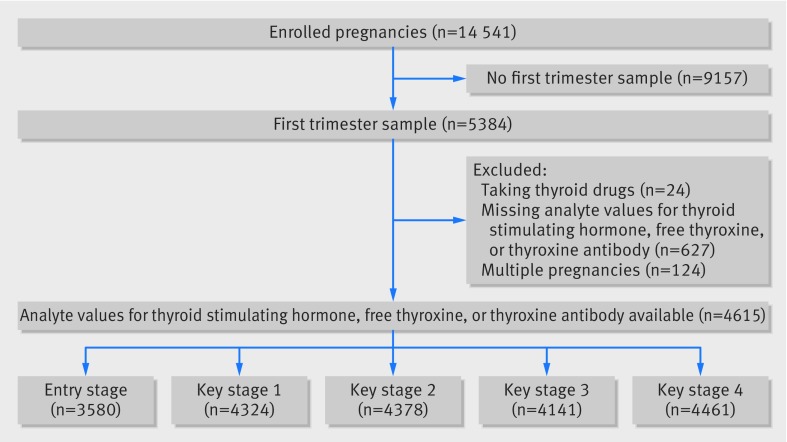
Study flow. Participants may have had more than one exclusion

The Avon Longitudinal Study of Parents and Children is a prospective birth cohort study investigating the health and development of children. The study has been described in full elsewhere.^33^ ^34^ ^35^ Women with expected delivery dates between 1 April 1991 and 31 December 1992 were eligible for recruitment. Ethical approval was obtained from the Avon Longitudinal Study of Parents and Children’s Law and Ethics Committee and from the National Health Service local ethics committee. Figure 1 shows that a total of 14 541 women were enrolled and privides the numbers excluded for missing data, mothers treated with thyroid related drugs during the index pregnancy, multiple pregnancies, or with pre-existing thyroid disease**. **Supplementary table 1 shows the baseline characteristics of the entire Avon Longitudinal Study of Parents and Children cohort and those who did and did not have thyroid function assessed.

### Thyroid function tests

Thyroid stimulating hormone levels, free thyroxine, and thyroid peroxidase anitbodies were assessed on stored serum samples using an Abbott Architect i2000 immunoassay analyser with functional sensitivity of 0.05 mIU/L or less. Interassay and intra-assay coefficients of variation were less than 5% for all analytes. The limit of detection for thyroid stimulating hormone was 0.005 mIU/L. The limit of detection for free thyroxine was 0.3 pmol/L. A thyroid peroxidase antibody titre of 6 IU/mL or more was classed as positive, in accordance with UK National External Quality Assessment Service data. For thyroid peroxidase antibody negative women the 2.5th to 97.5th centile for thyroid stimulating hormone levels was 0.070 mIU/L to 2.55 mIU/L, and the 2.5th to 97.5th centile for free thyroxine levels was 12.53 pmol/L to 22.78 pmol/L (0.97 ng/dL to 1.77 ng/dL).

We categorized women into clinical groups according to thyroid stimulating hormone and free thyroxine levels in the first trimester as defined by below the 2.5th and above the 97.5th centile in thyroid peroxidase antibody negative women; hypothyroid (thyroid stimulating hormone >97.5th and free thyroxine <2.5th); subclinical hypothyroid (>97.5th and 2.5th to 97.5th); isolated hypothyroxinaemia (2.5th to 97.5th and <2.5th); isolated hyperthyroxinaemia (2.5th to 97.5th and >97.5th); subclinical hyperthyroid (<2.5th and 2.5th to 97.5); and hyperthyroid (<2.5th and >97.5th) (supplementary table 2). Additional sensitivity analyses were performed for hypothyroidism as defined by a thyroid stimulating hormone level above 10 mIU/L irrespective of free thyroxine level.^22^ ^23^ ^36^ Supplementary sensitivity analyses were performed with clinical categorisation according to thyroid stimulating hormone and free thyroxine gestational age-specific reference ranges in an iodine replete population.^37^ As recognised clinical categories were small in number, we performed further sensitivity analyses by combining hypothyroidism, subclinical hypothyroidism, and isolated hypothyroxinaemia categories to represent reduced maternal thyroid function (hypothyroid combined). Isolated hyperthyroxinaemia, subclinical hyperthyroidism, and hyperthyroidism were combined to represent increased maternal thyroid function (hyperthyroid combined).

### Academic outcomes

#### Key stages assessment

A data linkage was conducted with the Department for Education educational attainment data provided for children included in the Avon Longitudinal Study of Parents and Children who attend publicly funded schools. In England, Wales and Northern Ireland, students aged between 4 and 16 follow a national curriculum which is organised into blocks of years called key stages, undertaken at specific ages (supplementary table 3). Baseline assessment of language, reading, writing, mathematics, social, problem solving, small motor, and large motor skills are performed before starting school education (entry stage). At the end of each key stage, the child’s performance is formally assessed as part of the program of National Curriculum Tests, which cover English, mathematics, and science. The final assessment (key stage 4) is the externally marked General Certificate of Secondary Education (GCSE) examination, which is the principal means of assessing educational attainment at the end of compulsory secondary education. Students study up to 12 subjects for GCSE. Subjects are graded individually on a scale of A* (highest) to G (lowest). Universities and employers usually regard A* to C as pass grades. For entry to further education, and apprenticeship schemes, the usual requirement would be five GCSE passes at A* to C, including both English and mathematics. These GCSE and preceding key stage scores are considered to be a true measure of academic performance.

Scores from entry stage were individually examined for each of the eight domains, with individual subject and overall scores assessed for key stage 1 to key stage 3. For key stage 2 and key stage 3, scores were available individually for English, mathematics, and science. The GCSE results were examined in three ways. First, we assessed the absolute number of GCSE passed at A* and A (any *v* none), passed (grades A* to C), and attempted (grades A* to G). Second, we utilised a GCSE score awarding a minimum of 16 points for a G grade with an additional 6 points for each grade increase, resulting in a maximum of 58 points for an A* grade. This was in turn analysed as either a total score or more usually a capped score summed over the eight best GCSE grades achieved.^38^ This capped score avoided conflating the number of GCSEs taken and the grades achieved and enabled analysis of effects across the full distribution of student ability. Lastly, we considered attainment of both GCSE and General National Vocational Qualifications (GNVQ), with scoring of GNVQ grades in a similar manner to GCSE grades and consideration of the total GCSE or GNVQ continuous score.

### Statistical analysis

Spearman's correlation coefficient was used to determine the association between thyroid measures. Thyroid stimulating hormone and thyroid peroxidase antibody were log-transformed and were approximately normally distributed with these transformations. For our main analyses, we examined both the continuous associations of thyroid stimulating hormone, free thyroxine, and thyroid peroxidase antibody and the effect of each clinical categories with educational outcomes. Linearity was checked using restricted cubic splines. Possible departure from linear associations between thyroid function measures and education outcomes were explored by adding quadratic terms to the model. We assessed the effect of clinical categories compared with euthyroid, using multivariable regression. Modified Park’s tests were used to determine the appropriate regression models (gamma, Poisson, and logistic) for discrete GCSE outcomes. We also assessed the effect of maternal thyroid function on an individual’s repeat educational outcomes scores. Total scores from key stages 1, 2, and 3, and capped GCSE scores were standardised by subtracting the mean and dividing by the standard deviation within each score. Linear mixed effects models were fitted with time as a fixed effect and a random effect of subject. In all of our primary analyses we adjusted for the following key confounders: maternal age, parity, ethnicity, body mass index, smoking status, alcohol consumption prepregnancy (none, fewer than one glass per week, or more than one glass per week), alcohol consumption during pregnancy (none, fewer than one glass per week, more than one glass per week), maternal social class, maternal education, and paternal education. In a sensitivity analyses we additionally adjusted for the following potential mediators: birthweight, child sex, child head circumference at birth, gestational age at birth, and mode of delivery. Of the 3580 to 4461 mothers with complete thyroid function and education data, there were some participants with missing data on key confounders (supplementary table 4). This varied from 72 (1.5%) for maternal age to 1361 (29.4%) for paternal education. Missing values for covariates were imputed using multivariate multiple imputation, with five switching cycles and producing 100 imputation datasets. The regression models were performed on each imputed dataset and then the results were pooled using Rubin’s rules.^39^ ^40^ The distributions of observed and imputed variables were similar (supplementary table 5). In this paper we present results from the imputed datasets. The results from those with complete data (n=1242 to 1952) are presented in supplementary table 1 for comparison. All analyses were performed with R version 3.1.3 or above, with packages: MICE version 2.22, nlme version 3.1-131, ggplot2 version 0.9.3.1, and gplots version 2.3-45.

### Patient involvement

No patients were involved in setting the research question or the outcome measures, nor were they involved in developing plans for recruitment, design, or implementation of the study. No patients were asked to advise on interpretation or writing up of results. Research findings from the Avon Longitudinal Study of Parents and Children are reported on the study website and disseminated to participants as part of mailed newsletters, email updates, and an annual participant meeting.

## Results

Figure 1 shows that of the original 14 541 recruited to the Avon Longitudinal Study of Parents and Children cohort, 5384 had first trimester samples available for analysis, with thyroid stimulating hormone, free thyroxine, and thyroid peroxidase antibody status available for 4615 mothers. Thyroid function tests were performed on samples taken at a median of 10 weeks gestation (interquartile range 8-12 weeks) with 34 (0.74%) women newly identified as having hypothyroidism, 166 (3.6%) subclinical hypothyroidism, 93 (2.0%) isolated****hypothyroxinaemia, 57 (1.2%) subclinical hyperthyroidism, and 40 (0.87%) hyperthyroidism based on the criteria we have used. An elevated thyroid peroxidase antibody was detected in 469 (10.2%) women. As anticipated, thyroid stimulating hormone and free thyroxine were negatively correlated (r −0.29, 95% confidence interval −0.30 to −0.25), with thyroid stimulating hormone positively associated with thyroid peroxidase antibody titre (0.16, 0.13 to 0.18). Of the 4614 women, 61.8% of women with hypothyroidism and 49.4% with subclinical hypothyroidism had an elevated thyroid peroxidase antibody, as compared with 8.2% with euthyroidism and 12.5% with hyperthyroidism. Table 1 shows the baseline thyroid function tests for each of the clinical categories, with the respective maternal and pregnancy characteristics presented in supplementary table 4.

**Table 1:** First trimester thyroid function tests for each clinical category. Values are medians (interquartile range) unless stated otherwise

Characteristic	Hypothyroidism (n=34)	Subclinical hypothyroidism (n=166)	Isolated hypothyroxinaemia (n=93)	Euthyroidism (n=4169)	Isolated hyperthyroxinaemia (n=55)	Subclinical hyperthyroidism (n=57)	Hyperthyroidism (n=40)
Thyroid stimulating hormone (mIU/L)	5.12 (2.94-13.62)	3.22 (2.78-3.95)	1.16 (0.89-1.53)	0.97 (0.64-1.38)	0.80 (0.25-2.18)	0.04 (0.02-0.05)	0.02 (0.01-0.03)
Free thyroxine (pmol/L)	11.46 (9.09-12.08)	15.30 (14.13-16.59)	11.88 (11.18-12.29)	16.21 (14.91-17.62)	25.08 (23.47-27.17)	19.30 (18.05-20.87)	26.26 (23.68-28.64)
Thyroid peroxidase antibody (IU/mL)	63.16 (2.95-471.50)	5.69 (2.02-143.40)	1.90 (1.40-3.04)	1.92 (1.34-2.97)	1.66 (0.81-3.06)	2.23 (1.63-3.34)	2.23 (1.42-3.04)
No (%) of elevated thyroid peroxidase antibody*	21 (62)	82 (49)	6 (6)	343 (8)	6 (11)	6 (10)	5 (12)

* Titre ≥6 IU/mL was classed as elevated.

### Analysis using continuous data

Figure 1 shows that educational outcomes were available at school entry stage for 3580 children, at key stage 1 for 4324, at key stage 2 for 4378, at key stage 3 for 4141, and at key stage 4 for 4461, with results available at all five time points for 3011 children. Table 2 shows that there was no strong evidence of a clinically important association of maternal thyroid stimulating hormone and free thyroxine with overall entry stage assessment score or Standard Assessment Test summary scores at any of the three key stages assessed. Similarly, we did not find evidence of important associations between maternal thyroid stimulating hormone and free thyroxine with the total number of GCSEs attempted, number passed, or grade at which they were passed (table 2). The same results were foundwhen continuous scores of GCSE or General National Vocational Qualifications attainment were assessed (supplementary table 5). Analysis of the individual components of the entry stage assessment did not show any association of maternal thyroid stimulating hormone, free thyroxine, or thyroid peroxidase antibody titre with language, reading, writing, mathematics, social, problem solving, small motor, or large motor skills (supplementary table 6). For both key stages 2 and 3 there were no associations of maternal thyroid function with English, mathematics, or science scores (supplementary table 7). There was an isolated positive association of thyroid peroxidase antibody titre with key stage 1 score, but associations of thyroid peroxidase antibody titre with cognitive outcome at any of the other stages or any of the individual component scores were very close to the null. Associations of maternal thyroid stimulating hormone, free thyroxine, and thyroid peroxidase antibody with the educational outcomes were all close to the null value in the repeat measures analysis (supplementary table 8).

**Table 2:** Association of first trimester maternal thyroid function with cognitive outcomes. Values are regression coefficients (95% confidence intervals) unless otherwise stated

Characteristic	Entry stage	Key stage 1	Key stage 2	Key stage 3		Key stage 4 No of GCSEs at grade		
						A* to A (OR)	A to C (RR)	A* to G (RGM)
N_obs_ (N_miss_)†	3580 (1035)	4324 (291)	4378 (237)	4141 (474)		NA	4461 (154)	NA
Age at assessment (years)	4.5 (4.2 to 4.8)	7.3 (7.1 to 7.7)	11.2 (10.9 to 11.5)	14.1 (13.8 to 14.4)		NA	15 (15 to 15)	NA
Thyroid stimulating hormone	0.00 (−0.07 to 0.08)	0.07 (−0.25 to 0.39)	0.10 (−1.16 to 1.35)	1.05 (−1.16 to 3.27)		0.87 (0.70 to 1.07)	0.98 (0.94 to 1.02)	1.00 (0.97 to 1.03)
Free thyroxine	0.00 (−0.01 to 0.01)	0.01 (−0.03 to 0.05)	−0.03 (−0.18 to 0.13)	−0.08 (−0.35 to 0.19)		1.01 (0.99 to 1.04)	1.00 (1.00 to 1.01)	1.00 (1.00 to 1.00)
Thyroid peroxidase antibody	0.01 (−0.01 to 0.03)	0.01 (0.01 to 0.21)	0.09 (−0.48 to 0.29)	0.28 (−0.42 to 0.97)		0.98 (0.92 to 1.05)	1.00 (0.99 to 1.01)	1.00 (0.99 to 1.01)
Clinical categories:								
Hypothyroid	−0.06 (−0.37 to 0.24)	−0.30 (−1.58 to 0.98)	−1.97 (−7.11 to 3.16)	0.66 (−8.33 to 9.65)		0.25 (0.05 to 1.17)	0.95 (0.80 to 1.12)	1.04 (0.92 to 1.18)
Subclinical hypothyroid	0.16 (0.02 to 0.31)	0.73 (0.13 to 1.32)	0.99 (−1.29 to 3.27)	2.63 (−1.52 to 6.79)		1.02 (0.58 to 1.77)	1.02 (0.95 to 1.10)	1.02 (0.97 to 1.08)
Isolated hypothyroxinaemia	−0.04 (−0.23 to 0.16)	−0.55 (−1.32 to 0.22)	−2.47 (−5.57 to 0.62)	−5.46 (−11.04 to 0.13)		0.81 (0.48 to 1.37)	0.96 (0.87 to 1.06)	1.00 (0.93 to 1.08)
Isolated hyperthyroxinaemia	0.06 (−0.18 to 0.29)	−0.58 (−1.54 to 0.39)	−1.66 (−5.61 to 2.28)	−7.06 (−13.89 to −0.22)		0.92 (0.50 to 1.70)	0.91 (0.80 to 1.02)	0.91 (0.83 to 1.00)
Subclinical hyperthyroidism	0.01 (−0.27 to 0.28)	−0.45 (−1.46 to 0.55)	−1.03 (−5.08 to 3.02)	−2.15 (−9.34 to 5.03)		1.06 (0.55 to 2.04)	0.91 (0.80 to 1.04)	0.91 (0.83 to 1.01)
Hyperthyroidism	0.03 (−0.25 to 0.31)	0.25 (−0.91 to 1.41)	0.60 (−3.96 to 5.17)	−1.47 (−9.30 to 6.36)		0.98 (0.45 to 2.11)	1.08 (0.94 to 1.23)	1.07 (0.96 to 1.19)

GCSEs=General Certificates of Secondary Education; OR=odds ratio; RR=rate ratio; RGM=ratio of geometric means; NA=not available.

† Number of observation (number missing).

### Analysis using clinical categories

**Fig 2:**
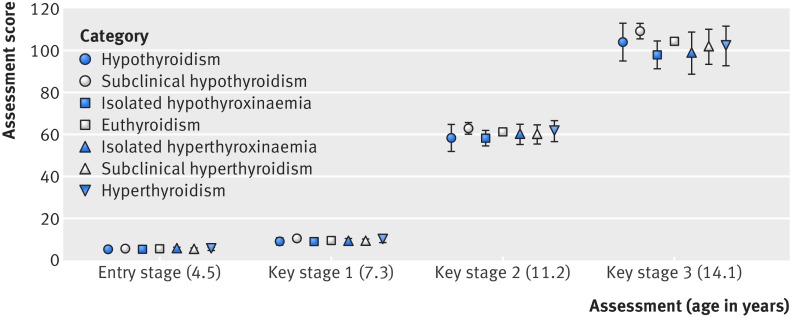
Mean assessment scores (95% confidence interval) relative to first trimester clinical categories of thyroid function

**Fig 3:**
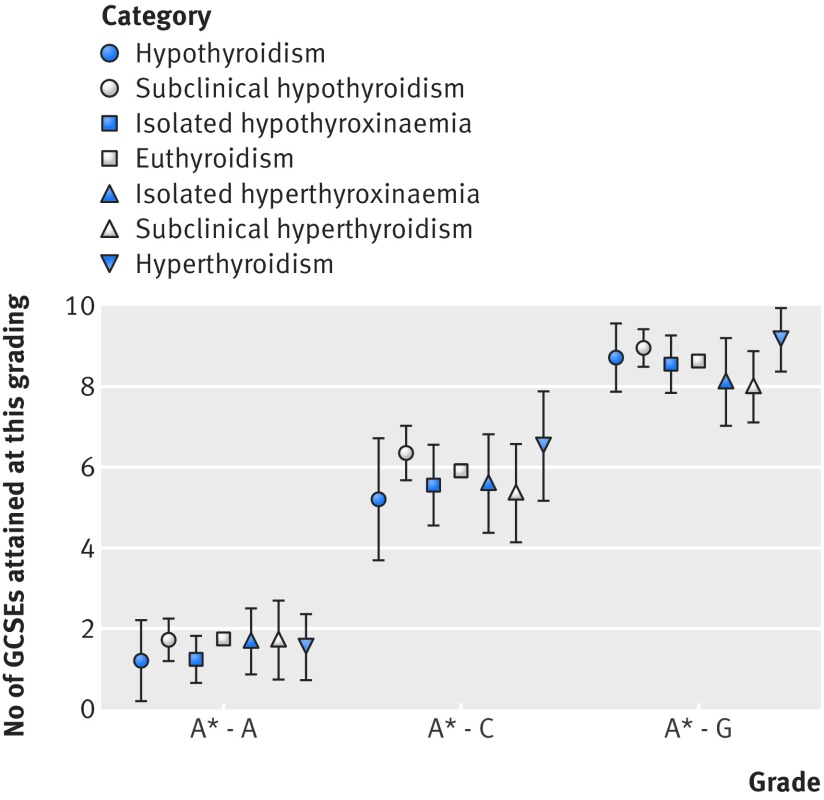
Mean number of General Certificates of Secondary Education attainment (95% confidence interval) relative to first trimester clinical categories of thyroid function. Median age at assessment was 15 years (interquartile range 15-15)

Figure 2 shows that children of mothers who had undiagnosed hypothyroidism, subclinical hypothyroidism, isolated hypothyroxinaemia, isolated hyperthyroxinaemia, subclinical hyperthyroidism, or hyperthyroidism during pregnancy did not achieve different entry stage scores as compared with children of mothers with euthyroidism. Similarly, assessment scores for each stage did not differ between the categories of maternal thyroid function (fig 2). Figure 3 shows that assessment of GCSE attainment did not demonstrate any differences between clinical categories of maternal thyroid function. Similar results were found when GCSEs were expressed as continuous, capped, and binary outcomes. Similar results were obtained when we restricted hypothyroidism to women with thyroid stimulating hormone levels above 10mIU/L (n=17 women) or categorised according to iodine replete reference ranges or collapsed clinical categories (see supplementary tables 11 and 12). The lack of association for individual and collapsed clinical categories was robust to adjustment for confounders and potential mediators irrespective of the gestational age specific reference ranges used for categorisation.

Findings from the complete case analyses were largely consistent with those from the imputation datasets, though with wider confidence intervals (supplementary tables 9 and 10). Findings were also similar when we adjusted for measured confounders and potential mediators (supplementary tables 13 to 15).

## Discussion

Clinical and epidemiological studies have previously suggested that maternal thyroid dysfunction in early pregnancy is associated with a range of adverse child outcomes.^22^ ^23^ The most important of these is the potential contribution of maternal hypothyroidism to poor cognitive functioning in infancy and childhood.^2^ ^5^ ^7^ ^8^ ^9^ ^41^ In this prospective longitudinal study we did not observe association of maternal first trimester thyroid function with long term educational attainment or on any of the preceding school performance assessment stages. We were able to examine the associations of thyroid stimulating hormone, free thyroxine, and thyroid peroxidase antibody titre with meaningful educational outcomes. We examined thyroid measures both on a continuous scale and also in women who would have had a new diagnosis of hypothyroidism and hyperthyroidism if routine thyroid function testing had been performed. For all of these individual measures, and for concealed diagnoses of thyroid function, there was no consistent evidence of association with child educational attainment.

### Comparison with other studies

Animal studies have shown that maternal hypothyroxinaemia in the first half of pregnancy can alter neurogenesis and lead to neuronal migration errors in the developing brain.^42^ Observational studies investigating the effect of low antenatal maternal free thyroxine concentrations on childhood cognitive functioning have reported inconsistent results and were of variable size (n=220 to 5049).^2^ ^9^ ^43^ ^44^ ^45^ ^46^ ^47^ Severe maternal hypothyroxinaemia (free thyroxine <5th centile, n=11 of 220) was associated with a reduction of 14.1 points (95% confidence interval 5.9 to 22.0) in the Psychomotor Bayley Scales of Infant Development (BSID) at 10 months of age but not the mental BSID.^43^ A second study assessing 1761 children reported a reduction of 3.4 (95% confidence interval 0.02 to 6.67) in the mental BSID at 14 months in the child of mothers with a free thyroxine below the 5th centile (n=82 of 1643) but the psychomotor BSID was unaffected.^46^ A third study reported that maternal free thyroxine below the 20th centile (n=57 of 287) was associated with a reduction in the Brunet–Lézine scale at 18 months of age.^48^ The most recent study assessing 3839 children suggested an inverted u shaped association between maternal free thyroxine and intelligence quotient at age six, with low and high free thyroxine associated with 1.4-3.8 point reduction in child intelligence quotient.^2^ These studies were limited by assessment at one age. When repeat assessments were performed at 18 months and 30 months (n=2926), maternal hypothyroxinaemia was associated with an increased risk of expressive language delay at both time points, and an increased risk of nonverbal cognitive delay at 30 months.^45^ Conversely, other studies with longer follow up have not found an association between maternal hypothyroxinaemia and cognitive outcomes on repeat assessments at 6 months and 3 years (n=500),^44^ or at 6, 12, 24, and 60 months (n=287).^47^ The largest study to date (n=5049) did not observe any differences in scholastic performance at age eight when children of mothers with thyroid dysfunction were compared with children of mothers with euthyroidism.^9^ At age 16 when the adolescents evaluated their own scholastic performance by questionnaire, adolescents of mothers with subclinical hypothyroidism and hyperthyroidism had higher adjusted odds of having self reported difficulties in mathematics, but no strong evidence for an association was observed for isolated hypothyroxinaemia.^9^ Distinct from previous series,^2^ addition of quadratic terms or use of repeat measures did not suggest a more complex relation of thyroid measures with educational outcomes.

The first of three randomised controlled trials on the effect of antenatal screening and treatment of mild thyroid dysfunction did not show a difference in childhood intelligence quotient at three years between children of treated (n=390) and untreated mothers (n=404)^21^. Post hoc analysis separately testing the effect of levothyroxine treatment on cognitive functioning in children of mothers with low free thyroxine levels only, also revealed no difference in outcomes.^21^ Similarly, screening and treatment of subclinical hypothyroidism (n=339 treated, n=338 placebo) or isolated hypothyroxinaemia (265, 261) was not effective in improving neurodevelopmental outcomes through to age five years.^20^ The discordance between the smaller observational studies, the current study, and the randomised controlled trials data showing no effect of maternal thyroid dysfunction on child cognitive outcomes may reflect the small size of the earlier observational studies, possible publication bias, lack of adjustment for maternal confounders in those earlier studies, and use of different assessment tools, with even the widely used BSID being poorly related to later cognitive and educational outcomes.^49^ Heterogeneous criteria used to define normal levels of thyroid stimulating hormone and free thyroxine during the first trimester may also contribute, with the threshold for defining normal thyroid stimulating hormone values varying from 2.2 mU/L to 4.8 mU/L.^43^ ^45^ ^48^ We purposely did not utilise external reference ranges, as these may be assay specific, with current guidelines recommending derivation of local trimester specific reference ranges. This addresses the physiological changes in pregnancy that influence thyroid function, including increased renal iodine excretion, increased thyroxine binding globulin, decreased albumin concentrations, increased thyroid hormone production, and the thyroid stimulatory effects of human chorionic gonadotrophin.^23^ ^36^ We excluded multiple pregnancies owing to their increased human chorionic gonadotrophin levels and performed additional sensitivity analyses using reference ranges specific to gestational age from an iodine replete population and the results were unchanged.

### Strengths and weaknesses of this study

This is the first study linking maternal thyroid function to longer term and meaningful educational attainment. Our study has several strengths, including size, duration of follow-up, robust analyses including imputation to enable adjustment for confounders, use of an assay which has been validated for long term stored samples,^50^ consideration of an unscreened population incorporating undiagnosed maternal hypothyroidism and hyperthyroidism, and use of a national validated educational database to minimise loss of outcome data or bias owing to participation in a follow-up research clinic. 

We acknowledge several limitations. Maternal samples were not available for the whole Avon Longitudinal Study of Parents and Children cohort, however, this would only bias results if mothers with available samples were systematically different from those without an available maternal sample, which they were not. Long term storage of maternal samples has been associated with an increase in thyroid peroxidase antibody titre but not that of thyroid stimulating hormone or free thyroxine.^50^ Our results were similar when we analysed our data continuously or when we derived clinical categories independent of thyroid peroxidase antibody status and just used thyroid stimulating hormone and free thyroxine concentrations. We acknowledge that we did not have repeat thyroid function tests across gestation, and were unable to examine whether patterns of thyroid stimulating hormone and free thyroxine across gestation contribute to educational attainment.^51^ Similarly, maternal iodine status was only available for 436 women with first trimester thyroid function data, limiting assessment of its contribution to long term educational outcomes. 

The national pupil database of government maintained establishments has 89.5% coverage of all English students nationally and 84.3% in the area of the Avon Longitudinal Study of Parents and Children cohort. We anticipate that key stage information was not available from the national pupil database owing to students living outside England or with insufficient information for linkage. As special care establishments are government maintained we do not anticipate that affected individuals have been lost to follow-up, similarly sixth form and further education colleges all contribute to the national pupil database ensuring maximal data coverage. Private schools do not contribute to the national pupil database, however, inclusion of privately educated students may be expected to attenuate any association owing to the extra support and improved GCSE results observed for these schools. 

We recognise that we assessed multiple associations and the isolated positive association of thyroid peroxidase antibody titre and key stage 1 scores may reflect a chance finding, particularly as there was no consistent association with performance at any other age of assessment. We were unable to assess subtle effects of maternal thyroid dysfunction on individual aspects of cognitive function such as executive functioning, memory, visual attention, and sensorimotor development. We would suggest the overall impact would be potentially small as there was no clinically important impact on academic achievement at any age. We acknowledge that family and parental factors, children's dietary patterns and thyroid function, and education may have attenuated to the null any potential detrimental effect of maternal thyroid function on cognitive outcomes, but this would further support that maternal thyroid dysfunction does not have permanent consequences for long term child intellectual development.

### Conclusion

Our results suggest that maternal thyroid dysfunction during early pregnancy is unlikely to be an important determinant of poor educational attainment in children.

**What is already known on this topic**

Case-control and cohort studies have suggested that maternal thyroid dysfunction, and low levels of maternal thyroid hormone in particular, in the first trimester are associated with lower intellectual function and impaired cognitive processes in exposed children
Two randomised controlled trials of levothyroxine therapy in newly diagnosed mothers with hypothyroidism have not observed any decrement in cognitive function at age 3 or 5 compared compared with untreated mothers
Current clinical guidelines recommend treatment for mothers with thyroid dysfunction as long term intellectual effects are unknown

**What this study adds**

Our study shows that maternal thyroid stimulating hormone levels and free thyroxine concentrations in the first trimester are not associated with performance in national curricular assessments from age 54 months to age 15 years
Women with thyroid dysfunction in pregnancy can be reassured that their thyroid disease will not impact their child’s school performance or educational attainment

## Web Extra

Extra material supplied by the author

## Notes

Ethical approval: This research has the approval of the Avon Longitudinal Study of Parents and Children Ethics and Law Committee and associated governance procedures.

Data sharing: The technical appendix and statistical code are available from the corresponding author at scott.nelson@glasgow.ac.uk. Data are available in accordance with the Avon Longitudinal Study of Parents and Children access policy (http://www.bristol.ac.uk/alspac/researchers/access/).

Transparency: The lead author (SMN) affirms that the manuscript is an honest, accurate, and transparent account of the study being reported; that no important aspects of the study have been omitted; and that any discrepancies from the study as planned have been explained.
