# Supplementary material for: Maternal thyroid function and child educational attainment: prospective cohort study
Source: BMJ. 2018 Feb 20;360:k452. doi: 10.1136/bmj.k452 (PMC5819484; doi:10.1136/bmj.k452)
Supplement: Supplementary file 1 — Appendix: Supplementary materials [file nels040304.ww1.pdf]

Maternal thyroid function and offspring educational attainment: prospective cohort study in the United Kingdom

Scott M Nelson PhD1, Caroline Haig PhD2, Alex McConnachie PhD2, Naveed Sattar FRCP (Glas)3, Susan M Ring PhD4,5, George Davey Smith PhD4,5, Debbie A Lawlor PhD4,5, Robert S Lindsay FRCP (Glas)3

Supplemental information

Supplemental Table 1 Characteristics of entire ALSPAC cohort with missing and non-missing thyroid information.

Variable			All subjects	Missing	Non-missing
Age (years)	-	N_{OBS} (N_{MISS})	14082 (1363)	8776 (1285)	5306 (78)
		Mean (SD)	28.0 (5.0)	27.91 (5.06)	28.15 (4.80)
		Median (IQR)	[illegible]8.0 (25.0, 31.0)	[illegible]0 (25.00, 31.00)	[illegible]0 (25.00, 31.00)
		[Range]	[15.0, 44.0]	[illegible]15.00, 44.00]	[illegible]15.00, 44.00]
Smoking during pregnancy		N_{OBS} (N_{MISS})	13364 (2081)	8327 (1734)	5037 (347)
	No	N (%)	9883 (74.0%)	[illegible]107 (73.3%)	3776 (75.0%)
	Quit1st	N (%)	808 (6.0%)	529 (6.4%)	279 (5.5%)
	Yes	N (%)	2673 (20.0%)	1691 (20.3%)	982 (19.5%)
BMI pre pregnancy	-	N_{OBS} (N_{MISS})	11671 (3774)	7177 (2884)	4494 (890)
		Mean (SD)	22.9 (3.8)	22.95 (3.92)	22.88 (3.69)
		Median (IQR)	[illegible]2.2 (20.5, 24.4)	[illegible].6 (20.47, 24.43)	[illegible].7 (20.47, 24.38)
		[Range]	[12.5, 54.7]	[illegible]12.50, 54.66]	[illegible]14.23, 47.36]
BMI category		N_{OBS} (N_{MISS})	11671 (3774)	7177 (2884)	4494 (890)
	< 18.5	N (%)	582 (5.0%)	382 (5.3%)	200 (4.5%)
	18.5 - 24.99	N (%)	8695 (74.5%)	[illegible]291 (73.7%)	3404 (75.7%)
	25 - 29.99	N (%)	1755 (15.0%)	1092 (15.2%)	663 (14.8%)
	30+	N (%)	639 (5.5%)	412 (5.7%)	227 (5.1%)
Alcohol pre-pregnancy		N_{OBS} (N_{MISS})	13238 (2207)	8227 (1834)	5011 (373)
	never	N (%)	1117 (8.4%)	742 (9.0%)	375 (7.5%)
	less than 1 glass per wk	N (%)	4989 (37.7%)	3075 (37.4%)	1914 (38.2%)
	1 glass per wk	N (%)	5655 (42.7%)	3499 (42.5%)	[illegible]156 (43.0%)
	1-2 glasses per day	N (%)	1255 (9.5%)	774 (9.4%)	481 (9.6%)
	3+ per day	N (%)	222 (1.7%)	137 (1.7%)	85 (1.7%)
Alcohol at 12 weeks		N_{OBS} (N_{MISS})	13200 (2245)	8206 (1855)	4994 (390)
	never	N (%)	6004 (45.5%)	3702 (45.1%)	[illegible]302 (46.1%)
	less than 1 glass per wk	N (%)	5107 (38.7%)	3127 (38.1%)	1980 (39.6%)
	1 glass per wk	N (%)	1835 (13.9%)	1200 (14.6%)	635 (12.7%)
	1+ per day	N (%)	254 (1.9%)	177 (2.2%)	77 (1.5%)

Variable			All subjects	Missing	Non-missing
Socioeconomic position		N_{OBS} (N_{MISS})	10125 (5320)	6197 (3864)	3928 (1456)
	I	N (%)	597 (5.9%)	383 (6.2%)	214 (5.4%)
	II	N (%)	3185 (31.5%)	2000 (32.3%)	1185 (30.2%)
	III non manual	N (%)	4329 (42.8%)	2533 (40.9%)	1796 (45.7%)
	III manual	N (%)	791 (7.8%)	511 (8.2%)	280 (7.1%)
	IV	N (%)	997 (9.8%)	625 (10.1%)	372 (9.5%)
	V	N (%)	222 (2.2%)	143 (2.3%)	79 (2.0%)
	Armed forces	N (%)	4 (0.0%)	2 (0.0%)	2 (0.1%)
Maternal education		N_{OBS} (N_{MISS})	12499 (2946)	7715 (2346)	4784 (600)
	CSE or None	N (%)	2527 (20.2%)	1599 (20.7%)	928 (19.4%)
	Vocational	N (%)	1229 (9.8%)	741 (9.6%)	488 (10.2%)
	0 level	N (%)	4329 (34.6%)	2603 (33.7%)	1726 (36.1%)
	A level	N (%)	2804 (22.4%)	1740 (22.6%)	1064 (22.2%)
	Degree	N (%)	1610 (12.9%)	1032 (13.4%)	578 (12.1%)
Paternal education		N_{OBS} (N_{MISS})	9928 (5517)	6134 (3927)	3794 (1590)
	CSE	N (%)	2164 (21.8%)	1359 (22.2%)	805 (21.2%)
	Vocational	N (%)	818 (8.2%)	489 (8.0%)	329 (8.7%)
	O level	N (%)	2231 (22.5%)	1355 (22.1%)	876 (23.1%)
	A level	N (%)	2777 (28.0%)	1715 (28.0%)	1062 (28.0%)
	Degree	N (%)	1938 (19.5%)	1216 (19.8%)	722 (19.0%)
Ethnicity		N_{OBS} (N_{MISS})	12156 (3289)	7474 (2587)	4682 (702)
	White	N (%)	11543 (95.0%)	7063 (94.5%)	4480 (95.7%)
	Nonwhite	N (%)	613 (5.0%)	411 (5.5%)	202 (4.3%)
Parity		N_{OBS} (N_{MISS})	13129 (2316)	8169 (1892)	4960 (424)
	0	N (%)	5875 (44.7%)	3624 (44.4%)	2251 (45.4%)
	1	N (%)	4588 (34.9%)	2862 (35.0%)	1726 (34.8%)
	2	N (%)	1884 (14.3%)	1166 (14.3%)	718 (14.5%)
	3	N (%)	546 (4.2%)	359 (4.4%)	187 (3.8%)
	4+	N (%)	236 (1.8%)	158 (1.9%)	78 (1.6%)

Variable			All subjects	Missing	Non-missing
Pre-existing hypertension		N_{OBS} (N_{MISS})	12184 (3261)	7496 (2565)	4688 (696)
	No	N (%)	11737 (96.3%)	7232 (96.5%)	4505 (96.1%)
	Yes	N (%)	447 (3.7%)	264 (3.5%)	183 (3.9%)
Gestational hypertension		N_{OBS} (N_{MISS})	13580 (1865)	8457 (1604)	5123 (261)
	No	N (%)	11597 (85.4%)	7210 (85.3%)	4387 (85.6%)
	Yes	N (%)	1983 (14.6%)	1247 (14.7%)	736 (14.4%)
Diabetes glycosoria		N_{OBS} (N_{MISS})	13886 (1559)	8660 (1401)	5226 (158)
	Yes	N (%)	115 (0.8%)	80 (0.9%)	35 (0.7%)
	No	N (%)	13771 (99.2%)	8580 (99.1%)	5191 (99.3%)
Pre-term		N_{OBS} (N_{MISS})	8486 (6959)	5436 (4625)	3050 (2334)
	Yes	N (%)	858 (10.1%)	596 (11.0%)	262 (8.6%)
	No	N (%)	7628 (89.9%)	4840 (89.0%)	2788 (91.4%)
Gestation at delivery	-	N_{OBS} (N_{MISS})	14619 (826)	9309 (752)	5310 (74)
		Mean (SD)	38.4 (5.5)	37.76 (6.66)	39.43 (1.94)
		Median (IQR)	40.0 (38.0, 41.0)	40.00 (38.00, 41.00)	40.00 (39.00, 41.00)
		[Range]	[4.0, 47.0]	[4.00, 47.00]	[18.00, 44.00]
Birthweight (g)	-	N_{OBS} (N_{MISS})	13901 (1544)	8663 (1398)	5238 (146)
		Mean (SD)	3381.5 (580.8)	3362.50 (601.46)	3412.94 (543.63)
		Median (IQR)	3410.0 (3080.0, 3740.0)	3400.00 (3060.0, 3740.0)	3420.0 (3100.0, 3760.0)
		[Range]	[200.0, 5640.0]	[200.0, 5640.0]	[600.0, 5050.0]

N_{OBS} (N_{MISS}) Number of observations (Number of missing observations)

Supplemental Table 2: Classification of thyroid function

		TSH		
		<2.5th percentile	2.5th – 97.5th percentile	>97.5th percentile
fT4	<2.5th percentile	-	Isolated hypothyroxinaemia	Hypothyroid
	2.5th – 97.5th percentile	Subclinical hyperthyroidism	Euthyroid	Subclinical hypothyroid
	>97.5th percentile	Hyperthyroidism	Isolated hyperthyroxinaemia	-

Supplemental Table 3: National curriculum key stages and assessment

Age	Key Stage (KS)	Assessment
4 – 5 years	Entry Stage	National assessment of language, reading, writing, mathematics, social skills, problem solving, small motor skills, large motor skills. Individual and overall scores reported.
6 – 7 years	KS1	Assessment in English, maths and science and accumulated into overall score
7 – 8 years	KS2	Local teacher assessment
8 – 9 years	KS2	Local teacher assessment
9 – 10 years	KS2	Local teacher assessment
10 – 11 years	KS2	National assessments in English, maths and science with individual subject and overall score reported
11 – 12 years	KS3	Local teacher assessment
12 – 13 years	KS3	Local teacher assessment
13 – 14 years	KS3	National assessments in English, maths and science with individual subject and overall score reported
14 – 15 years	KS4	Some children take GCSEs
15 – 16 years	KS4	Most children take GCSEs or other national qualifications

Supplemental Table 4: Baseline maternal and pregnancy outcome characteristics for each clinical category

Characteristic	Statistic	Hypothyroid (N=34)	Subclinical hypothyroid (N=166)	Isolated hypo-thyroxinaemia (N=93)	Normal (N=4169)	Isolated hyper-thyroxinaemia (N=55)	Subclinical hyper-thyroidism (N=57)	Hyper-thyroidism (N=40)
Age (years)	N_{OBS} (N_{MISS})	4615 (0)	4615 (0)	4615 (0)	4615 (0)	4615 (0)	4615 (0)	4615 (0)
	Mean (SD)	28 (5)	29 (5)	29 (5)	28 (5)	29 (4)	29 (5)	29 (4)
Smoking during	N_{OBS} (N_{MISS})	30 (4)	160 (6)	88 (5)	3883 (286)	52 (3)	54 (3)	38 (2)
pregnancy	N (%)	18 (60)	131 (82)	59 (67)	2911 (75)	40 (77)	46 (85)	32 (84)
No	N (%)	2 (7)	11 (7)	4 (5)	208 (5)	3 (6)	1 (2)	3 (8)
Stopped during first trimester	N (%)	10 (3)	18 (11)	25 (28)	764 (20)	9 (17)	7 (13)	3 (8)
Yes								
Pre pregnancy BMI (kg/m^2)	Mean (SD)	24.4 (4.2)	23.6 (4.4)	23.1 (3.8)	22.8 (3.7)	22.7 (3.6)	22.8 (3.8)	22.7 (2.6)
Alcohol consumption pre-pregnancy	N_{OBS} (N_{MISS})	29 (5)	106 (60)	87 (6)	3861 (308)	52 (3)	54 (3)	38 (2)
Never	N (%)	1 (3.4)	8 (5.0)	6 (6.9)	293 (7.6)	0 (0.0)	9 (16.7)	6 (15.8)
Fewer than 1 glass per week	N (%)	15 (51.7)	59 (36.9)	27 (31.0)	1480 (38.3)	22 (42.3)	26 (48.1)	17 (44.7)
1 or more glasses per week	N (%)	13 (44.8)	93 (58.1)	54 (62.1)	2088 (54.1)	30 (57.7)	19 (35.2)	15 (39.5)
Alcohol consumption at 12 weeks	N_{OBS} (N_{MISS})	30 (4)	160 (6)	87 (6)	3846 (323)	52 (3)	54 (3)	38 (2)
Never	N (%)	15 (50.0)	80 (50.0)	35 (40.2)	1773 (46.1)	26 (50.0)	31 (57.4)	21 (55.3)
Fewer than 1 glass		9 (30.0)	54 (33.8)	39 (44.8)	1532 (39.8)	21 (40.4)	17 (31.5)	12 (31.6)

Characteristic	Statistic	Hypothyroid<br><br>(N=34)	Subclinical hypothyroid<br><br>(N=166)	Isolated hypo-thyroxinaemia<br><br>(N=93)	Normal<br><br>(N=4169)	Isolated hyper-thyroxinaemia<br><br>(N=55)	Subclinical hyper-thyroidism<br><br>(N=57)	Hyper-thyroidism<br><br>(N=40)
per week	N (%)							
1 or more glasses per week	N (%)	6 (20.0)	26 (16.2)	13 (14.9)	541 (14.1)	5 (9.6)	6 (11.1)	5 (13.2)
Socioeconomic	N_{OBS} (N_{MISS})	23 (11)	134 (32)	70 (23)	3025 (1144)	43 (12)	40 (17)	32 (8)
position	N (%)	1 (4.3)	10 (7.5)	3 (4.3)	166 (5.5)	2 (4.7)	3 (7.5)	2 (6.2)
I	N (%)	7 (30.4)	37 (27.6)	17 (24.3)	911 (30.1)	17 (39.5)	7 (17.5)	8 (25.0)
II	N (%)	10 (43.5)	69 (51.5)	38 (54.3)	1375 (45.5)	18 (41.9)	23 (57.5)	17 (53.1)
III non-manual	N (%)	1 (4.3)	7 (5.2)	5 (7.1)	221 (7.3)	3 (7.0)	2 (5.0)	1 (3.1)
III manual	N (%)	4 (17.4)	11 (8.2)	7 (10.0)	352 (11.6)	3 (7.0)	5 (12.5)	4 (12.5)
IV or V								
Parity	N_{OBS} (N_{MISS})	28 (6)	159 (7)	86 (7)	3827 (342)	52 (3)	54 (3)	38 (2)
0	N (%)	12 (42.9)	81 (50.9)	34 (39.5)	1780 (46.5)	24 (46.2)	15 (27.8)	9 (23.7)
1	N (%)	9 (32.1)	49 (30.8)	31 (36.0)	1308 (34.2)	15 (28.8)	23 (42.6)	20 (52.6)
2	N (%)	3 (10.7)	21 (13.2)	13 (15.1)	532 (13.9)	11 (21.2)	13 (24.1)	7 (18.4)
3+	N (%)	4 (14.3)	8 (5.0)	8 (9.3)	207 (5.4)	2 (3.8)	3 (5.6)	2 (5.3)
Maternal education	N_{OBS} (N_{MISS})	28 (6)	157 (9)	85 (8)	3684 (485)	53 (2)	54 (3)	37 (3)
CSE or None	N (%)	5 (17.9)	28 (17.8)	13 (15.3)	722 (19.6)	8 (15.1)	10 (18.5)	6 (16.2)
Vocational	N (%)	4 (14.3)	11 (7.0)	12 (14.1)	380 (10.3)	7 (13.2)	7 (13.0)	4 (10.8)
0 level	N (%)	11 (39.3)	55 (35.0)	37 (43.5)	1311 (35.6)	22 (41.5)	26 (48.1)	14 (37.8)
A level	N (%)	7 (25.0)	45 (28.7)	11 (12.9)	827 (22.4)	11 (20.8)	4 (7.4)	7 (18.9)
Degree	N (%)	1 (3.6)	18 (11.5)	12 (14.1)	444 (12.1)	5 (9.4)	7 (13.0)	6 (16.2)
Paternal education	N_{OBS} (N_{MISS})	28 (6)	157 (9)	85 (8)	3684 (485)	53 (2)	54 (3)	37 (3)
CSE	N (%)	5 (17.9)	28 (17.8)	13 (15.3)	722 (19.6)	8 (15.1)	10 (18.5)	6 (16.2)
Vocational	N (%)	4 (14.3)	11 (7.0)	12 (14.1)	380 (10.3)	7 (13.2)	7 (13.0)	4 (10.8)
O level	N (%)	11 (39.3)	55 (35.0)	37 (43.5)	1311 (35.6)	22 (41.5)	26 (48.1)	14 (37.8)
A level	N (%)	7 (25.0)	45 (28.7)	11 (12.9)	827 (22.4)	11 (20.8)	4 (7.4)	7 (18.9)
Degree	N (%)	1 (3.6)	18 (11.5)	12 (14.1)	444 (12.1)	5 (9.4)	7 (13.0)	6 (16.2)

Characteristic	Statistic	Hypothyroid (N=34)	Subclinical hypothyroid (N=166)	Isolated hypo-thyroxinaemia (N=93)	Normal (N=4169)	Isolated hyper-thyroxinaemia (N=55)	Subclinical hyper-thyroidism (N=57)	Hyper-thyroidism (N=40)
Ethnicity	N_{OBS} (N_{MISS})	28 (6)	155 (11)	85 (8)	3607 (562)	49 (6)	52 (5)	37 (3)
White	N (%)	27 (96.4)	151 (97.4)	81 (95.3)	3453 (95.7)	47 (95.9)	47 (90.4)	36 (97.3)
Nonwhite	N (%)	1 (3.6)	4 (2.6)	4 (4.7)	154 (4.3)	2 (4.1)	5 (9.6)	1 (2.7)
Offspring sex	N_{OBS} (N_{MISS})	34 (0)	166 (0)	93 (0)	4169 (0)	55 (0)	57 (0)	40 (0)
Male	N (%)	13 (38.2)	84 (50.6)	43 (46.2)	2172 (52.1)	24 (43.6)	28 (49.1)	17 (42.5)
Female	N (%)	21 (61.8)	82 (49.4)	50 (53.8)	1997 (47.9)	31 (56.4)	29 (50.9)	23 (57.5)
Gestation at delivery (weeks)	Median (IQR)	40.0 (39.0, 41.0)	40.0 (39.0, 41.0)	40.0 (39.0, 41.0)	40.0 (39.0, 41.0)	40.0 (39.0, 41.0)	40.0 (38.0, 41.0)	39.0 (38.0, 40.0)
Delivery method	N_{OBS} (N_{MISS})	33 (1)	159 (7)	86 (7)	3987 (182)	54 (3)	56 (1)	39 (1)
Assisted breech	N (%)	0 (0.0)	2 (1.3)	1 (1.2)	55 (1.4)	1 (1.9)	1 (1.8)	2 (5.1)
Caesarean section	N (%)	5 (15.2)	18 (11.3)	8 (9.3)	399 (10.0)	6 (11.1)	8 (14.3)	6 (15.4)
Forceps	N (%)	3 (9.1)	9 (5.7)	3 (3.5)	219 (5.5)	2 (3.7)	3 (5.4)	2 (5.1)
Other	N (%)	0 (0.0)	1 (0.6)	1 (1.2)	42 (1.1)	2 (3.7)	1 (1.8)	0 (0.0)
Spontaneous	N (%)	23 (69.7)	115 (72.3)	70 (81.4)	3033 (76.1)	41 (75.9)	42 (75.0)	29 (74.4)
Vacuum extraction	N (%)	2 (6.1)	14 (8.8)	3 (3.5)	239 (6.0)	2 (3.7)	1 (1.8)	0 (0.0)
Birthweight (g)	Median (IQR)	3470 (3268, 3725)	3580 (3200, 3865)	3400 (3125, 3789)	3430 (3120, 3760)	3448 (3125, 3655)	3630 (3250, 3760)	3360 (2890, 3708)
Head circumference (cm)	Median (IQR)	34.5 (34.0, 35.2)	35.1 (34.1, 36.1)	34.9 (34.0, 36.0)	34.9 (34.0, 35.8)	34.8 (34.1, 35.6)	35.0 (33.8, 35.7)	34.9 (33.8, 35.7)

N_{OBS} (N_{MISS}) Number of observations (Number of missing observations)

Data are N (%), mean (SD) or median (interquartile range).

Supplemental Table 5: Characteristics of complete and multiply imputed datasets

Characteristic	Nmiss (%)	Complete-case	Multiply imputed data
Age (years)	72 (1.5%)	28.06 (4.8)	28.06 (4.84)
Smoking during pregnancy	309 (6.5%)		
No		3237 (75.2%)	3450 (74.8%)
Stopped during first trimester		233 (5.4%)	255 (5.5%)
Yes		836 (19.4%)	910 (19.7%)
Pre pregnancy BMI (kg/m^2)	774 (16.7%)	22.9 (3.7)	22.91 (3.74)
Alcohol consumption pre-pregnancy	333 (7.2%)		
Never		323 (7.5%)	356 (7.7%)
Fewer than 1 glass per week		1647 (38.5%)	1755 (38.5%)
1 or more glasses per week		2312 (54.0%)	2483 (53.8%)
Alcohol consumption at 12 weeks	347 (7.5%)		
Never		1982 (46.4%)	2154 (46.6%)
Fewer than 1 glass per week		1684 (39.5%)	1812 (39.3%)
1 or more glasses per week		602 (14.1%)	649 (14.1%)
Socioeconomic position	1247 (27.1%)		
I		187 (4.8%)	224 (4.8%)
II		1004 (29.8%)	1266 (27.4%)
III non-manual		1551 (46.1%)	2102 (45.6%)
III manual		240 (7.1%)	369 (8.0%)
IV or V		386 (11.5%)	654 (14.2%)
Parity	423 (7.9%)		
0		1956 (46.1%)	2139 (46.3%)
1		1455 (34.3%)	1572 (34.1%)

Characteristic	Nmiss (%)	Complete-case	Multiply imputed data
2		600 (14.1%)	649 (14.1%)
3+		234 (5.5%)	255 (5.5%)
Maternal education	516 (11.1%)		
CSE or None		792 (19.3%)	924 (20.0%)
Vocational		425 (10.4%)	490 (10.6%)
0 level		1477 (36.0%)	1667 (36.1%)
A level		912 (22.2%)	1004 (21.8%)
Degree		493 (12.0%)	530 (11.5%)
Paternal education	1361 (29.4%)		
CSE		712 (21.9%)	1079 (23.4%)
Vocational		278 (8.5%)	410 (8.9%)
O level		750 (23.0%)	1070 (23.2%)
A level		900 (27.7%)	1257 (27.2%)
Degree		614 (18.9%)	799 (17.3%)
Ethnicity	601 (13.0%)		
White		3843 (95.7%)	4399 (95.3%)
Nonwhite		171 (4.3%)	216 (4.7%)
Offspring sex	1 (<0.001%)		
Male		2381 (51.6%)	2382 (51.6%)
Female		2233 (48.4%)	2233 (48.4%)
Gestation at delivery (weeks)	69 (1.5%)	39.5 (1.9)	39.5 (1.9)
Delivery method	184 (4.0%)		
Assisted breech		62 (1.4%)	65 (1.4%)
Caesarean section		451 (10.2%)	472 (10.2%)
Forceps		241 (5.5%)62	251(5.4%)
Other		(1.2%)	50 (1.1%)

Characteristic	Nmiss (%)	Complete-case	Multiply imputed data
Spontaneous<br>Vacuum extraction		4036 (75.3%)<br>261 (5.9%)	3505 (75.9%)<br>272 (5.9%)
Birthweight (g)	130 (2.8%)	3430 (532)	3427 (535)
Head circumference (cm)	1230 (26.6%)	34.9 (1.5)	34.8 (1.6)

Data are N (%), mean (SD)

Supplemental Table 6: Association of first trimester maternal thyroid function with GCSE outcomes

	**Total GCSE and equivalents ***	**Capped GCSE and equivalents ***	**Total GCSE/GNVQ***
TSH	-4.91<br>(-17.50 to 7.68)	-2.98<br>(-10.58 to 4.61)	-4.85<br>(-17.01 to 7.31)
fT_4	0.85<br>(-0.66 to 2.36)	0.58<br>(-0.33 to 1.49)	0.21<br>(-1.24 to 1.67)
TPO-Ab	1.76<br>(-2.14 to 5.66)	0.03<br>(-2.33 to 2.38)	-0.57<br>(-4.33 to 3.19)
**Clinical categories (referent to euthyroid)**			
Hypothyroid	-8.99<br>(-59.57 to 41.59)	2.48<br>(-28.05 to 33.00)	4.87<br>(-44.13 to 53.86)
Subclinical hypothyroid	2.59<br>(-20.87 to 26.06)	0.36<br>(-13.78 to 14.50)	2.56<br>(-20.15 to 25.26)
Isolated hypo-thyroxinaemia	-11.93<br>(-43.10 to 19.25)	-8.41<br>(-27.20 to 10.37)	-7.44<br>(-37.40 to 22.51)
Isolated hyperthyroxinaemia	-16.64<br>(-54.94 to 21.65)	-14.88<br>(-38.17 to 8.41)	-31.37<br>(-67.99 to 5.25)
Subclinical hyperthyroidism	-9.38<br>(-49.67 to 30.90)	-2.46<br>(-26.74 to 21.82)	-27.07<br>(-65.57 to 11.44)
Hyperthyroidism	23.96<br>(-21.65 to 69.56)	17.68<br>(-9.81 to 45.18)	18.31<br>(-25.15 to 61.77)

Values are β coefficients and 95% confidence intervals
GCSE score is awarded in range of 16 for grade G to 58 for Grade A for each GCSE with the "capped " score representing the composite of the best 8 GCSE's.

Model adjusts for maternal age, parity, ethnicity, body mass index, smoking status, alcohol consumption pre-pregnancy, alcohol consumption during pregnancy, maternal social class, , maternal education and paternal education.

Supplemental Table 7: Association of thyroid function with individual components of key stage assessments

	**TSH** (n = 4615)	**fT_4** (n = 4615)	**TPO-Ab titre** (n = 4615)
**Early stage assessment**			
Language	0.04 (-0.07 to 0.16)	-0.00 (-0.02 to 0.01)	0.01 (-0.02 to 0.05)
Reading	-0.00 (-0.09 to 0.08)	0.00 (-0.01 to 0.01)	0.01 (-0.02 to 0.04)
Writing	-0.01 (-0.10 to 0.08)	0.01 (-0.00 to 0.02)	0.01 (-0.02 to 0.03)
Maths	-0.01 (-0.12 to 0.10)	-0.00 (-0.01 to 0.01)	0.01 (-0.02 to 0.04)
**Key Stage 2**			
English	0.23 (-1.10 to 1.56)	0.01 (-0.15 to 0.17)	-0.05 (-0.46 to 0.36)
Maths	-0.07 (-1.90 to 1.76)	-0.03 (-0.25 to 0.19)	0.05 (-0.52 to 0.61)
Science	0.52 (-0.52 to 1.57)	-0.05 (-0.18 to 0.07)	-0.15 (-0.47 to 0.18)
**Key Stage 3**			
English	0.27 (-1.23 to 1.76)	0.02 (-0.16 to 0.20)	0.14 (-0.33 to 0.61)
Maths	-0.73 (-2.79 to 1.33)	0.05 (-0.20 to 0.30)	-0.25 (-0.90 to 0.39)
Science	0.23 (-2.15 to 2.61)	-0.00 (-0.29 to 0.29)	0.52 (-0.22 to 1.27)

Values are β coefficients and 95% confidence intervals

Model adjusts for maternal age, parity, ethnicity, body mass index, smoking status, alcohol consumption pre-pregnancy, alcohol consumption during pregnancy, maternal social class, maternal education and paternal education.

Supplemental Table 8: Association of thyroid function with repeat measures of educational outcomes from KS1 to GCSE

		Estimate*	95% CI	P-value
TSH (log) (n = 4615)	Univariate	0.037	(-0.043 to 0.118)	0.3634
	MV model 1	0.047	(-0.045 to 0.138)	0.3169
	MV model 2	0.081	(-0.023 to 0.187)	0.1267
fT4 (n = 4615)	Univariate	0.003	(-0.007 to 0.095)	0.5179
	MV model 1	-0.003	(-0.014 to 0.008)	0.6413
	MV model 2	-0.007	(-0.019 to 0.006)	0.3101
TPO-Ab (log) (n = 4615)	Univariate	0.020	(-0.006 to 0.044)	0.1336
	MV model 1	0.010	(-0.019 to 0.035)	0.5672
	MV model 2	0.012	(-0.020 to 0.044)	0.4654

Model 1 adjusts for maternal age, parity, ethnicity, body mass index, smoking status, alcohol consumption pre-pregnancy, alcohol consumption during pregnancy, maternal social class, maternal education and paternal education.
Model 2 additionally adjusts for birthweight, child sex, child head circumference at birth, gestational age at birth and mode of delivery
*KS1, KS2, KS3, capped GCSE all standardised and assessed by linear mixed effects model with time, and random effect of subject. Confidence intervals approximated using a normal approximation to the distribution of the restricted maximum likelihood estimators.

Supplemental Table 9: Association of first trimester maternal thyroid function with cognitive outcomes in complete cases

	Entry stage[1] (Linear regression coefficient)	Key Stage 1[1] (Linear regression coefficient)	Key Stage 2[1] (Linear regression coefficient)	Key Stage 3[1] (Linear regression coefficient)	Key Stage 4 Number of GCSEs at grade		
					A*-G[2] (RGM)	A-C[3] (RR)	Any A*-A[4] (OR)
N_{obs} with complete data	1522	1827	1953	1788		1952	
Age at assessment (years)	4.5 (4.2 to 4.8)	7.3 (7.1 to 7.7)	11.2 (10.9 to 11.5)	14.1 (13.8 to 14.4)		15 (15 to 15)	
TSH	0.04 (-0.06 to 0.15)	0.24 (-0.18 to 0.66)	0.80 (-0.80 to 2.40)	0.87 (-1.99 to 3.73)	1.00 (0.97 to 1.04)	0.99 (0.94 to 1.04)	0.98 (0.73, 1.31)
fT_4	-0.00 (-0.01 to 0.01)	-0.01 (-0.06 to 0.04)	-0.11 (-0.30 to 0.08)	-0.14 (-0.48 to 0.19)	1.00 (1.00 to 1.01)	1.00 (1.00 to 1.01)	1.00 (0.96, 1.03)
TPO-Ab	0.03 (-0.00 to 0.06)	0.09 (-0.03 to 0.22)	0.07 (-0.41 to 0.55)	-0.14 (-1.01 to 0.74)	0.99 (0.98 to 1.00)	1.00 (0.98 to 1.01)	0.96 (0.88, 1.05)
**Clinical categories (referent to euthyroid)**							
Hypothyroid	0.02 (-0.43 to 0.46)	0.47 (-1.26 to 2.20)	-0.14 (-7.14 to 6.87)	-3.60 (-16.14 to 8.93)	1.05 (0.91 to 1.22)	0.95 (0.76 to 1.19)	0.64 (0.18 to 2.36)
Subclinical hypothyroid	0.23 (0.05 to 0.41)	0.56 (-0.15 to 1.27)	0.19 (-2.52 to 2.90)	1.12 (-3.80 to 6.04)	0.98 (0.93 to 1.04)	0.97 (0.89 to 1.06)	0.66 (0.39 to 1.10)
Isolated hypo-thyroxinaemia	0.02 (-0.24 to 0.28)	-0.37 (-1.35 to 0.61)	-2.34 (-6.11 to 1.43)	-5.70 (-12.32 to 0.93)	1.00 (0.92 to 1.08)	0.95 (0.84 to 1.07)	0.62 (0.31 to 1.25)
Isolated hyperthyroxinaemia	-0.04 (-0.33 to 0.24)	-0.38 (-1.68 to 0.72)	-2.22 (-7.17 to 2.73)	-2.21 (-10.69 to 6.28)	1.01 (0.91 to 1.11)	1.03 (0.90 to 1.18)	1.12 (0.49 to 2.58)
Subclinical hyperthyroidism	0.10 (-0.27 to 0.46)	-0.38 (-1.74 to 0.99)	-1.33 (-6.52 to 3.85)	-3.57 (-12.87 to 5.74)	0.90 (0.81 to 1.01)	0.97 (0.83 to 1.14)	1.33 (0.54 to 3.30)
Hyperthyroidism	0.03 (-0.33 to 0.38)	-0.38 (-1.82 to 1.05)	-2.07 (-7.81 to 3.67)	-2.19 (-11.78 to 7.40)	1.02 (0.91 to 1.15)	1.00 (0.84 to 1.20)	0.70 (0.24 to 2.03)

N_{obs} number of observations for model with complete data on confounders.
For Key stage 4 N(obs) is for those where outcome data is available.
Model adjusts for maternal age, parity, ethnicity, body mass index, smoking status, alcohol consumption pre-pregnancy, alcohol consumption during pregnancy, maternal social class, , maternal education and paternal education (confounder model).
[1] Linear regression. Values are regression coefficients (β) and 95% confidence intervals.
[2] Gamma regression. Values are ratios of geometric means (RGM) and 95% confidence intervals.
[3] Poisson regression. Values are rate ratios (RR) and 95% confidence intervals.
[4] Logistic regression. Values are odds ratios (OR) and 95% confidence intervals. Outcome is <u>any</u> A*-A vs none.

Supplemental Table 10: Association of first trimester thyroid function with individual components of key stage assessments in complete cases

	**TSH**	**fT_4**	**TPO-Ab titre**
**Early stage assessment** (N_{obs} = 1522)			
Language	0.10 (-0.05 to 0.26)	-0.00 (-0.02 to 0.01)	0.04 (-0.01 to 0.09)
Reading	0.02 (-0.10 to 0.13)	-0.00 (-0.02 to 0.01)	0.03 (-0.01 to 0.06)
Writing	-0.02 (-0.14 to 0.10)	0.01 (-0.00 to 0.03)	0.01 (-0.03 to 0.04)
Maths	0.06 (-0.08 to 0.21)	-0.01 (-0.03 to 0.01)	0.04 (-0.01 to 0.08)
**Key Stage 2** (N_{obs} = 1990)			
English	1.28 (-0.46 to 3.02)	-0.09 (-0.29 to 0.12)	0.17 (-0.36 to 0.69)
Maths	1.07 (-1.33 to 3.48)	-0.19 (-0.47 to 0.10)	0.23 (-0.50 to 0.95)
Science	0.51 (-0.80 to 1.82)	-0.11 (-0.27 to 0.04)	-0.13 (-0.53 to 0.26)
**Key Stage 3** (N_{obs} = 1747)			
English	0.28 (-1.74 to 2.31)	-0.09 (-0.33 to 0.14)	0.23 (-0.39 to 0.86)
Maths	0.02 (-2.89 to 2.85)	-0.20 (-0.54 to 0.14)	-0.40 (-1.28 to 0.47)
Science	-0.42 (-3.62 to 2.77)	-0.13 (-0.51 to 0.24)	0.51 (-0.46 to 1.49)

Values are β coefficients and 95% confidence intervals.

Nobs number of observations for the model with complete data on confounders.
Model adjusts for maternal age, parity, ethnicity, body mass index, smoking status, alcohol consumption pre-pregnancy, alcohol consumption during pregnancy, maternal social class, maternal education and paternal education.

Supplemental Table 11: Association of first trimester maternal thyroid function with cognitive outcomes to using reference ranges for iodine replete population to define clinical categories.

	Entry stage[1]	Key Stage 1[1]	Key Stage 2[1]	Key Stage 3[1]	Key Stage 4 Number of GCSEs at grade		
					A*-G (RGM)[2]	A-C (RR)[3]	A*-A (OR)[4]
N_{obs} (N_{miss})	3580 (1035)	4324 (291)	4378 (237)	4141 (474)	4461 (154)		
Age at assessment (years)	4.5 (4.2 to 4.8)	7.3 (7.1 to 7.7)	11.2 (10.9 to 11.5)	14.1 (13.8 to 14.4)	15 (15 to 15)		
**Clinical categories (referent to euthyroid)**							
Hypothyroid	-0.28 (-0.76 to 0.19)	-0.96 (-2.90 to 0.97)	-6.39 (-14.31 to 1.54)	-4.82 (-17.87 to 8.22)	1.06 (0.88 to 1.28)	0.75 (0.55 to 1.02)	0.14 (0.02 to 1.19)
Subclinical hypothyroid	0.13 (-0.03 to 0.29)	0.79 (0.13 to 1.45)	1.91 (-0.67 to 4.48)	3.49 (-1.12 to 8.10)	1.03 (0.97 to 1.10)	1.08 (1.00 to 1.17)	0.95 (0.62 to 1.48)
Isolated hypo-thyroxinaemia	0.56 (-0.04 to 1.16)	0.03 (-2.18 to 2.24)	5.31 (-3.83 to 14.46)	6.80 (-8.90 to 22.50)	1.16 (0.92 to 1.45)	1.41 (1.09 to 1.82)	2.21 (0.50 to 9.82)
Isolated hyperthyroxinaemia	-0.04 (-0.11 to 0.04)	0.13 (-0.17 to 0.43)	0.06 (-1.12 to 1.23)	-1.15 (-3.21 to 0.92)	1.01 (0.98 to 1.04)	1.02 (0.98 to 1.06)	1.09 (0.89 to 1.33)
Subclinical hyperthyroidism	-0.38 (-0.76 to 0.00)	0.05 (-1.30 to 1.41)	-3.57 (-9.57 to 2.42)	-3.56 (-14.11 to 7.00)	0.83 (0.72 to 0.96)	0.82 (0.67 to 1.01)	1.40 (0.53 to 3.67)
Hyperthyroidism	0.17 (-0.02 to 0.35)	-0.06 (-0.81 to 0.69)	0.39 (-2.48 to 3.27)	0.56 (-4.51 to 5.62)	1.05 (0.97 to 1.13)	1.08 (0.99 to 1.19)	1.17 (0.72 to 1.92)

N_{obs} (N_{miss}) number of observation (number missing). For Key stage 4 N(obs) is for those where outcome data is available
Values are β coefficients and 95% confidence intervals.
Model adjusts for maternal age, parity, ethnicity, body mass index, smoking status, alcohol consumption pre-pregnancy, alcohol consumption during pregnancy, maternal social class, maternal education and paternal education.

Supplemental Table 12: Association of collapsed clinical categories of first trimester maternal thyroid function with cognitive outcomes.

	Entry stage[1]	Key Stage 1[1]	Key Stage 2[1]	Key Stage 3[1]	Key Stage 4 Number of GCSEs at grade		
					A*-G (RGM)[2]	A-C (RR)[3]	A*-A (OR)[4]
N_{obs} (N_{miss})	3580 (1035)	4324 (291)	4378 (237)	4141 (474)		4461 (154)	
Age at assessment (years)	4.5 (4.2 to 4.8)	7.3 (7.1 to 7.7)	11.2 (10.9 to 11.5)	14.1 (13.8 to 14.4)		15 (15 to 15)	
**Clinical categories (referent to euthyroid)**							
Hypo combined	0.07 (-0.04 to 0.18)	0.17 (-0.27 to 0.62)	-0.43 (-2.17 to 1.31)	0.02 (-3.12 to 3.15)	1.02 (0.97 to 1.06)	0.99 (0.94 to 1.05)	0.71 (0.52 to 0.97)
Hyper combined	0.03 (-0.12 to 0.18)	-0.31 (-0.91 to 0.28)	-0.79 (-3.19 to 1.60)	-3.83 (-8.00 to 0.34)	0.95 (0.90 to 1.01)	0.96 (0.89 to 1.03)	0.94 (0.63 to 1.40)

N_{obs} (N_{miss}) number of observation (number missing). For Key stage 4 N(obs) is for those where outcome data is available
Values are β coefficients and 95% confidence intervals
Model adjusts for maternal age, parity, ethnicity, body mass index, smoking status, alcohol consumption pre-pregnancy, alcohol consumption during pregnancy, maternal social class, maternal education and paternal education
[1] Linear regression. Values are regression coefficients (β) and 95% confidence intervals.
[2] Gamma regression. Values are ratios of geometric means (RGM) and 95% confidence intervals.
[3] Poisson regression. Values are rate ratios (RR) and 95% confidence intervals.
[4] Logistic regression. Values are odds ratios (OR) and 95% confidence intervals. Outcome is any A*-A vs none.

Supplemental Table 13: Association of first trimester maternal thyroid function with cognitive outcomes adjusting for confounders and mediators

	Entry stage[1]	Key Stage 1[1]	Key Stage 2[1]	Key Stage 3[1]	Key Stage 4 Number of GCSEs at grade		
					**A*-G (RGM)[2]**	**A-C (RR)[3]**	**A*-A (OR)[4]**
N_{obs} (N_{miss})	3580 (1035)	4324 (291)	4378 (237)	4141 (474)	4461 (154)		
Age at assessment (years)	4.5 (4.2 to 4.8)	7.3 (7.1 to 7.7)	11.2 (10.9 to 11.5)	14.1 (13.8 to 14.4)	15 (15 to 15)		
TSH	0.00 (-0.07 to 0.08)	0.07 (-0.24 to 0.38)	0.07 (-1.16 to 1.31)	0.99 (-1.18 to 3.17)	1.00 (0.97 to 1.03)	0.98 (0.94 to 1.02)	0.87 (0.70 to 1.08)
fT_4	0.00 (-0.01 to 0.01)	0.01 (-0.03 to 0.05)	-0.02 (-0.17 to 0.12)	-0.08 (-0.34 to 0.18)	1.00 (1.00 to 1.00)	1.00 (1.00 to 1.01)	1.01 (0.99 to 1.04)
TPO-Ab	0.01 (-0.02 to 0.03)	0.11 (0.01 to 0.20)	-0.10 (-0.48 to 0.28)	0.26 (-0.42 to 0.95)	1.00 (0.99 to 1.01)	1.00 (0.99 to 1.01)	0.98 (0.92 to 1.04)
**Clinical categories (referent to euthyroid)**							
Hypothyroid	0.14 (-0.00 to 0.29)	0.61 (0.03 to 1.20)	-1.75 (-6.81 to 3.30)	1.00 (-7.83 to 9.83)	1.06 (0.93 to 1.20)	0.95 (0.80 to 1.13)	0.51 (0.20 to 1.31)
Subclinical hypothyroid	-0.05 (-0.36 to 0.25)	-0.22 (-1.47 to 1.04)	0.87 (-1.39 to 3.12)	2.51 (-1.58 to 6.60)	1.02 (0.96 to 1.08)	1.01 (0.95 to 1.09)	0.73 (0.49 to 1.09)
Isolated hypo-thyroxinaemia	-0.04 (-0.23 to 0.15)	-0.54 (-1.30 to 0.22)	-2.40 (-5.45 to 0.65)	-4.94 (-10.44 to 0.56)	1.00 (0.92 to 1.08)	0.95 (0.86 to 1.05)	0.73 (0.43 to 1.25)
Isolated hyperthyroxinaemia	0.05 (-0.19 to 0.28)	-0.56 (-1.51 to 0.38)	--1.64 (-5.53 to 2.25)	-7.03 (-13.75 to -0.31)	0.91 (0.83 to 0.99)	0.91 (0.81 to 1.03)	0.85 (0.45 to 1.58)
Subclinical hyperthyroidism	0.00 (-0.27 to 0.27)	-0.51 (-1.49 to 0.48)	-0.94 (-4.93 to 3.05)	-1.86 (-8.93 to 5.22)	0.91 (0.83 to 1.01)	0.91 (0.80 to 1.03)	1.05 (0.54 to 2.03)
Hyperthyroidism	0.04 (-0.24 to 0.32)	0.37 (-0.76 to 1.51)	0.52 (-3.96 to 5.01)	-1.99 (-9.67 to 5.69	1.07 (0.96 to 1.20)	1.08 (0.94 to 1.24)	0.97 (0.45 to 2.10)

N_{obs} (N_{miss}) number of observation (number missing). For Key stage 4 N(obs) is for those where outcome data is available
Values are estimated mean change (β coefficients) and 95% confidence intervals
Model adjusts for maternal age, parity, ethnicity, body mass index, smoking status, alcohol consumption pre-pregnancy, alcohol consumption during pregnancy, maternal social class, maternal education and paternal education (confounders) and additionally for birthweight, child head circumference at birth, gestational age at birth and mode of delivery (potential mediators).
[1] Linear regression. Values are regression coefficients (β) and 95% confidence intervals.
[2] Gamma regression. Values are ratios of geometric means (RGM) and 95% confidence intervals.
[3] Poisson regression. Values are rate ratios (RR) and 95% confidence intervals.
[4] Logistic regression. Values are odds ratios (OR) and 95% confidence intervals. Outcome is any A*-A vs none.

Supplemental Table 14: Association of first trimester maternal thyroid function with GCSE outcomes adjusting for confounders and mediators

	**Total GCSE and equivalents ***	**Capped GCSE and equivalents ***	**Total GCSE/GNVQ***
TSH	-4.79 (-17.36 to 7.77)	-2.90 (-10.47 to 4.68)	-4.72 (-16.86 to 7.42)
fT_4	0.92 (-0.59 to 2.43)	0.60 (-0.31 to 1.51)	0.30 (-1.15 to 1.76)
TPO-Ab	1.76 (-2.13 to 5.66)	-0.01 (-2.36 to 2.34)	-0.55 (-4.31 to 3.21)
**Clinical categories (referent to euthyroid)**			
Hypothyroid	-7.66 (-58.18 to 42.87)	-2.46 (-43.20 to 38.29)	6.35 (-42.63 to 55.32)
Subclinical hypothyroid	1.07 (-22.37 to 24.51)	2.38 (-18.07 to 22.82)	1.33 (-21.35 to 24.02)
Isolated hypo-thyroxinaemia	-13.14 (-44.27 to 17.99)	-8.91 (-27.62 to 9.79)	-8.79 (-38.72 to 21.13)
Isolated hyperthyroxinaemia	-15.77 (-53.99 to 22.45)	-12.82 (-35.81 to 10.17)	-30.61 (-67.19 to 5.97)
Subclinical hyperthyroidism	-9.50 (-49.77 to 30.77)	-2.43 (-26.68 to 21.82)	-27.84 (-66.35 to 10.67)
Hyperthyroidism	25.92 (-19.68 to 71.52)	18.99 (-8.48 to 46.46)	19.23 (-24.20 to 62.66)

Values are β coefficients and 95% confidence intervals

GCSE score is awarded in range of 16 for grade G to 58 for Grade A for each GCSE with the “capped “ score representing the composite of the best 8 GCSE’s.

Model adjusts for maternal age, parity, ethnicity, body mass index, smoking status, alcohol consumption pre-pregnancy, alcohol consumption during pregnancy, maternal social class, maternal education and paternal education (confounders) and additionally for birthweight, child head circumference at birth, gestational age at birth and mode of delivery (potential mediators).

Supplemental Table 15: Association of thyroid function with individual components of key stage assessments adjusting for confounders and mediators

	**TSH** (n = 4615)	**fT_4** (n = 4615)	**TPO-Ab titre** (n = 4615)
**Early stage assessment**			
Language	0.04 (-0.07 to 0.16)	-0.00 (-0.02 to 0.01)	0.01 (-0.02 to 0.04)
Reading	0.00 (-0.01 to 0.01)	0.00 (-0.01 to 0.01)	0.01 (-0.02 to 0.04)
Writing	-0.01 (-0.10 to 0.08)	0.01 (-0.00 to 0.02)	0.01 (-0.02 to 0.03)
Maths	-0.01 (-0.12 to 0.09)	-0.00 (-0.01 to 0.01)	0.01 (-0.02 to 0.04)
**Key Stage 2**			
English	0.20 (-1.11 to 1.51)	0.01 (-0.15 to 0.17)	-0.06 (-0.47 to 0.34)
Maths	-0.12 (-1.93 to 1.68)	-0.03 (-0.25 to 0.19)	0.03 (-0.52 to 0.59)
Science	0.51 (-0.53 to 1.54)	-0.05 (-0.18 to 0.07)	-0.15 (-0.47 to 0.17)
**Key Stage 3**			
English	0.24 (-1.24 to 1.71)	0.01 (-0.16 to 0.19)	0.16 (-0.31 to 0.62)
Maths	-0.74 (-2.78 to 1.31)	0.05 (-0.20 to 0.29)	-0.28 (-0.92 to 0.36)
Science	0.11 (-2.25 to 2.48)	0.00 (-0.28 to 0.29)	0.51 (-0.23 to 1.24)

Values are β coefficients and 95% confidence intervals

Model adjusts for maternal age, parity, ethnicity, body mass index, smoking status, alcohol consumption pre-pregnancy, alcohol consumption during pregnancy, maternal social class, maternal education and paternal education (confounders) and additionally for birthweight, child head circumference at birth, gestational age at birth and mode of delivery (potential mediators).d
